# Causal null hypotheses of sustained treatment strategies: What can be tested with an instrumental variable?

**DOI:** 10.1007/s10654-018-0396-6

**Published:** 2018-05-02

**Authors:** Sonja A. Swanson, Jeremy Labrecque, Miguel A. Hernán

**Affiliations:** 1 Department of Epidemiology, Erasmus MC, P.O. Box 2040, 3000 CA Rotterdam, The Netherlands; 2 Department of Epidemiology, Harvard T. H. Chan School of Public Health, Boston, USA; 3 Department of Biostatistics, Harvard T. H. Chan School of Public Health, Boston, USA; 4 Harvard-MIT Division of Health Sciences and Technology, Boston, USA

**Keywords:** Instrumental variable, Mendelian randomization, Hypothesis testing, Causal null hypothesis

## Abstract

Sometimes instrumental variable methods are used to test whether a causal effect is null rather than to estimate the magnitude of a causal effect. However, when instrumental variable methods are applied to time-varying exposures, as in many Mendelian randomization studies, it is unclear what causal null hypothesis is tested. Here, we consider different versions of causal null hypotheses for time-varying exposures, show that the instrumental variable conditions alone are insufficient to test some of them, and describe additional assumptions that can be made to test a wider range of causal null hypotheses, including both sharp and average causal null hypotheses. Implications for interpretation and reporting of instrumental variable results are discussed.

**Electronic supplementary material:**

The online version of this article (10.1007/s10654-018-0396-6) contains supplementary material, which is available to authorized users.

Instrumental variables are often used in observational studies, e.g., many Mendelian randomization studies, to obtain numerical estimates of causal effects. The validity of the effect estimates requires two conditions: (i) the proposed instrumental variable is indeed an instrumental variable, or instrument, as formalized below, and (ii) an additional condition requiring either some form of effect homogeneity or a monotonic relation between the instrumental variable and the exposure [1]. Condition (ii) is untenable in many research settings and has been met with skepticism by some investigators [1–4]. Because condition (ii) is often questionable, an alternative is to change the goal of the analysis from obtaining a numerical estimate of the causal effect to simply determining whether the exposure has *any* effect on the outcome [5]. For the purposes of such causal null testing, it has been argued, condition (i) is sufficient [5, 6].

A difficulty with causal null hypothesis testing is that it is often unclear what the hypothesis is. For example, studying the effect of alcohol requires the specification of the effect in terms of a contrast of hypothetical interventions sustained over time, e.g., “consume a glass of red wine every other day throughout adulthood” [7, 8]. However, many studies using instrumental variable methods leave the strategies of interest unspecified. Because there are many such strategies, there are multiple possible contrasts and therefore multiple causal null hypotheses that can be tested.

Here, we consider different versions of causal null hypotheses, describe conditions under which the instrument-outcome association can be used to test these hypotheses, and discuss how to conduct and interpret results from these tests. We begin by reviewing established results in the simple setting of time-fixed treatments [9], and then extend our discussion to more realistic settings with time-varying treatments.

## Causal null hypotheses for time-fixed treatments

**Fig. 1:**
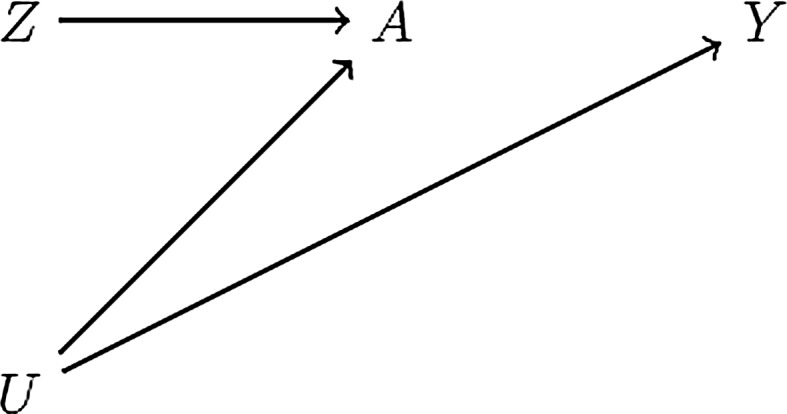
Causal diagram depicting a causal instrument *Z*, a time-fixed treatment *A*, an outcome *Y*, and unmeasured confounders *U*

The causal diagram in Fig. 1 depicts a causal instrument *Z*, a time-fixed treatment *A*, and an outcome *Y*. For simplicity, suppose both the instrument and the treatment are binary, the outcome is continuous, and faithfulness holds (for additional settings, see Appendix). An instrument-based test of the causal null hypothesis of treatment *A* is a test of whether the instrument-outcome association is null, that is, a test of the equality of the quantities ${\text{E}}[Y|Z = 1]$ and ${\text{E}}[Y|Z = 0]$. We now discuss the validity of this test for different types of causal null hypotheses.

First, let us first consider the sharp causal null hypothesis: treatment does not affect the outcome for any individual in the study population. Formally, $Y_{i}^{a = 1} = Y_{i}^{a = 0}$ for all individuals *i*, where $Y_{i}^{a}$ is individual *i*’s counterfactual or potential outcome under treatment level *a*. Because there is no arrow from *A* to *Y*, Fig. 1 represents a setting in which the sharp causal null holds.

In this setting, the quantities ${\text{E}}[Y|Z = 1]$ and ${\text{E}}[Y|Z = 0]$ are expected to be equal because, in Fig. 1, *Z* and *Y* are d-separated (for a proof based on counterfactual expressions see the Appendix). By the contrapositive, if our estimates of ${\text{E}}[Y|Z = 1]$ and ${\text{E}}[Y|Z = 0]$ in the study population are not equal and *Z* is an instrument, then we have evidence against the sharp causal null.

**Fig. 2:**
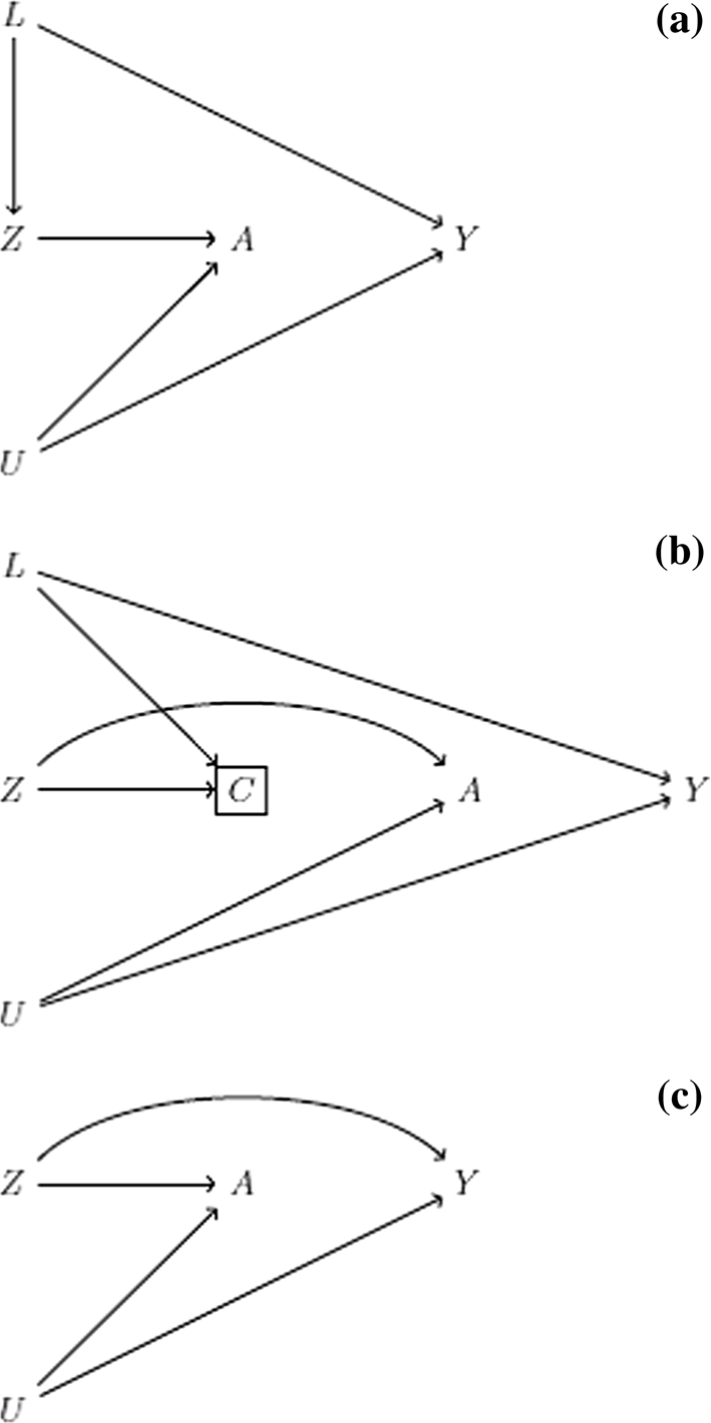
Causal diagrams depicting some violations of the instrumental conditions for a proposed instrument *Z*, a time-fixed treatment *A*, and an outcome *Y*. The scenarios represent **a** a violation of the instrumental exchageability condition via confounding, **b** a violation of the instrumental exchangeability condition via selection bias, and **c** a violation of the instrumental exclusion restriction  condition via a direct path from *Z* to *Y*. In (**a**, **b**), *Z* would satisfy the instrumental conditions conditional on *L*

Unfortunately, in real-world data analyses, we can never know for sure that* Z* is an instrument. If *Z* were not an instrument, as depicted in the causal diagrams of Fig. 2, then *Z* and *Y* are not d-separated even though the sharp causal null holds. Therefore, if our estimates of ${\text{E}}[Y|Z = 1]$ and ${\text{E}}[Y|Z = 0]$ in the study population are not equal, then we have evidence that at least one of the following is true: the sharp causal null does not hold or the proposed instrument is not an instrument. Note that under Fig. 2a, b, it would still be possible to find evidence against the sharp causal null if ${\text{E}}[Y|Z = 1,L = l]$ and ${\text{E}}[Y|Z = 0,L = l]$ were not equal in at least one stratum *L* = *l*; no such possibility exists for Fig. 2c.

Second, let us consider the average causal null hypothesis: treatment does not affect the average outcome in the study population, or ${\text{E}}\left[ {Y^{a = 1} } \right] = {\text{E}}\left[ {Y^{a = 0} } \right]$. The average causal null hypothesis is of interest when, for example, we plan to apply an intervention to an entire population. Because the sharp causal null hypothesis implies the average causal null hypothesis, the latter holds under the causal diagram in Fig. 1.

When the average causal null hypothesis holds, ${\text{E}}[Y|Z = 1]$ and ${\text{E}}[Y|Z = 0]$ are not guaranteed to be equal without additional conditions. One additional condition that would guarantee equality is a monotonic treatment effect: for a binary *A*, the treatment is either beneficial or harmful for all individuals in the study population (e.g., $Y_{i}^{a = 1} \le Y_{i}^{a = 0}$ for all individuals *i*) [9]. By the contrapositive, whenever our estimates of ${\text{E}}[Y|Z = 1]$ and ${\text{E}}[Y|Z = 0]$ are not equal, we have evidence that at least one of the following is true: the average causal null does not hold, the proposed instrument is not an instrument, or the treatment effect is not monotonic.

Finally, another causal null hypothesis is the “complier” average causal null hypothesis: ${\text{E}}\left[ {Y^{a = 1} |A^{z = 0} < A^{z = 1} } \right] = {\text{E}}[Y^{a = 0} |A^{z = 0} < A^{z = 1} ]$ [10]. Because this expression has no analogue for time-varying treatments, we do not consider it here.

Importantly, if testing were the only goal, we would not need the common instrumental variable analysis or related methods developed to estimate treatment effects. Yet, many published analyses focus on whether or not their results demonstrated a non-null causal effect, but use instrumental variable analyses and report numeric effect estimates. This is perhaps due to investigators recognizing the additional assumptions needed for these effect estimates to be valid are biologically implausible, let alone the additional issues with interpretation of these effect estimates when the treatment strategies are sustained over time [2, 7, 8, 11]. Therefore, if the investigators are reluctant to interpret the magnitude of the point estimate as that of an average causal effect, they can increase the transparency of their analysis by either making their reluctance explicit or restricting the presentation of results to the testing of the null.

## Causal null hypotheses for time-varying treatments

**Fig. 3:**
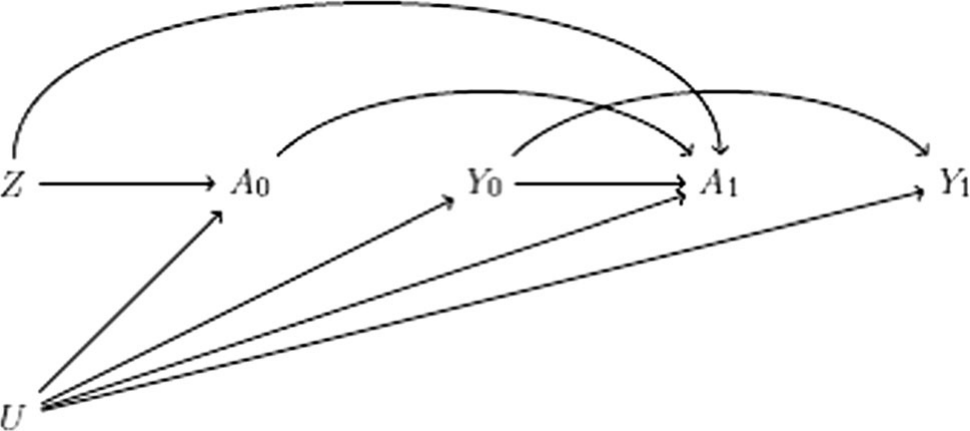
Causal diagram depicting a causal instrument *Z*, a treatment *A* measured at two time points (*A*_0_, *A*_1_), an outcome *Y* measured at two time points (*Y*_0_, *Y*_1_), and unmeasured confounders *U*

The causal diagram in Fig. 3 depicts a binary instrument *Z*, a time-varying treatment measured at two time points (*A*_0_, *A*_1_), and an outcome measured at two time points (*Y*_0_, *Y*_1_). The causal null hypothesis of interest is now concerning whether the joint effect of the time-varying treatment (*A*_0_, *A*_1_) on the outcome *Y*_1_ is null. (Results for the effect of *A*_0_ on *Y*_0_ follow immediately from the time-fixed treatment setting discussed above.) An instrument-based test of the joint causal null hypothesis of treatment (*A*_0_, *A*_1_) is a test of the equality of the quantities ${\text{E}}[Y_{1} |Z = 1]$ and ${\text{E}}[Y_{1} |Z = 0]$. We now discuss the validity of this test for different types of causal null hypotheses.

First, consider the joint sharp causal null hypothesis: treatment *at any time* does not affect the outcome for any individual in the study population. Formally, $Y_{1i}^{{a_{0} = 1,a_{1} = 1}} = Y_{1i}^{{a_{0} = 0,a_{1} = 1}} = Y_{1i}^{{a_{0} = 1,a_{1} = 0}} = Y_{1i}^{{a_{0} = 0,a_{1} = 0}}$ for all individuals *i*. Because there is no arrow from either *A*_0_ or *A*_1_ to *Y*_1_, Fig. 3 represents a setting in which the joint sharp causal null hypothesis holds.

When the joint sharp causal null holds and *Z* is a true instrument, then the quantities ${\text{E}}[Y_{1} |Z = 1]$ and ${\text{E}}[Y_{1} |Z = 0]$ are equal because, in Fig. 3, *Z* and $Y_{1}$ are d-separated. By the contrapositive, if our estimates of ${\text{E}}[Y_{1} |Z = 1]$ and ${\text{E}}[Y_{1} |Z = 0]$ are not equal and *Z* is an instrument, then we have evidence against the joint sharp causal null hypothesis. However, knowing that ${\text{E}}[Y_{1} |Z = 1]$ and ${\text{E}}[Y_{1} |Z = 0]$ are not equal does not inform which of the four counterfactual outcomes are not equal: even if *Z* were an instrument, we have evidence that at least one of these counterfactual outcomes is not equal to the others for at least one individual, but we would not have evidence against a sharp causal null comparing only (for example) continuous treatment [$Y_{1i}^{{a_{0} = 1,a_{1} = 1}}$] and continuous non-treatment [$Y_{1i}^{{a_{0} = 0,a_{1} = 0}}$].

Second, consider the joint average causal null hypothesis: treatment does not affect the average outcome in the study population, or ${\text{E}}\left[ {Y_{1}^{{a_{0} = 1,a_{1} = 1}} } \right] = {\text{E}}\left[ {Y_{1}^{{a_{0} = 0,a_{1} = 1}} } \right] = {\text{E}}\left[ {Y_{1}^{{a_{0} = 1,a_{1} = 0}} } \right] = {\text{E}}\left[ {Y_{1}^{{a_{0} = 0,a_{1} = 0}} } \right].$ As for a time-fixed treatment, our observed data on ${\text{E}}[Y_{1} |Z = 1]$ and ${\text{E}}[Y_{1} |Z = 0]$ provides no evidence for or against the average causal null without further assumptions. We can extend the monotonic treatment effect condition above to time-varying treatments: for example, specifying $Y_{1i}^{{a_{0} = 1,a_{1} = 1}}$ is the minimum value and $Y_{1i}^{{a_{0} = 0,a_{1} = 0}}$ is the maximum value across all possible outcomes for each individual *i* in the study population (see Appendix for more general expressions of a monotonic treatment effect condition). Under this additional condition, then we indeed would expect the quantities to be equal. By the contrapositive, this implies that whenever our estimates of ${\text{E}}[Y_{1} |Z = 1]$ and ${\text{E}}[Y_{1} |Z = 0]$ are not equal, we have evidence that at least one of the following is true: the joint average causal null does not hold, the proposed instrument is not an instrument, or the treatment effect is not monotonic.

Finally, we could also consider causal null hypotheses regarding the effect of *A*_1_ on *Y*_1_ without reference to the earlier treatment time *A*_0_: a sharp causal null hypothesis of $Y_{1i}^{{a_{1} = 1}} = Y_{1i}^{{a_{1} = 0}}$ for all individuals *i,* and an average causal null hypothesis of ${\text{E}}\left[ {Y_{1}^{{a_{1} = 1}} } \right] = {\text{E}}\left[ {Y_{1}^{{a_{1} = 0}} } \right]$. For estimating ${\text{E}}[Y_{1} |Z = 1]$ and ${\text{E}}[Y_{1} |Z = 0]$ to provide evidence for or against either of these conditions, however, there cannot be a path from *Z* to *A*_0_ to *Y*_1_ (either directly or through *Y*_0_). In other words, *Z* needs to satisfy the instrumental conditions (i) for *A*_1_ by itself, not necessarily jointly for *A*_0_ and *A*_1_. A similar line of reasoning applies to considerations of causal null hypotheses related to the effect of *A*_0_ on *Y*_1_.

These observations are summarized in Table 1 and formalized in the Appendix. Specifically, the Appendix covers results for non-binary instruments and treatments (including continuous treatments, as are common for Mendelian randomization studies), provides proofs that also apply to non-causal instruments [1, 2], and extends the above observations to an arbitrary number of treatment and outcome times.

**Table 1:** Conclusions about causal null hypotheses under the assumptions encoded in the causal diagram in Fig. 3

Causal null hypothesis	Null association between instrument and outcome	Non-null association between instrument and outcome
*Sharp causal null*		
*A*_0_ on *Y*_0_	No evidence against	Evidence against
(*A*_0_, *A*_1_) on *Y*_1_	No evidence against	Evidence against
*A*_0_ on *Y*_1_	No evidence against	No evidence against
*A*_1_ on *Y*_1_	No evidence against	No evidence against
*Average causal null*		
*A*_0_ on *Y*_0_	No evidence against	If monotonic treatment effect, evidence against
(*A*_0_, *A*_1_) on *Y*_1_	No evidence against	If monotonic treatment effect, evidence against
*A*_0_ on *Y*_1_	No evidence against	No evidence against
*A*_1_ on *Y*_1_	No evidence against	No evidence against

## On evidence regarding the direction and magnitude of causal effects

Suppose we find evidence against a causal null hypothesis and accept condition (i) that the proposed instrument is indeed an instrument. Can we infer the direction and magnitude of the average causal effect without making the homogeneity conditions (ii) that lead to point identification? (Estimation of average causal effects under homogeneity assumptions is discussed at length elsewhere [1, 11–13].)

If we find that ${\text{E}}\left[ {Y_{1} |Z = 1} \right] \ne {\text{E}}\left[ {Y_{1} |Z = 0} \right]$ then, assuming *Z* is an instrument, we have no information about the direction or the size of an effect. This observation has been made for time-fixed treatments [9]; here we extend it to time-varying treatments as well.

Using only the instrumental conditions (i), we can compute bounds for the average causal effect. If both the lower and upper bound were on the same side of the null (i.e., both positive or both negative) we would identify the direction of the average causal effect. However, in most practical settings, the lower and upper bounds straddle the null and thus do not identify the direction of the effect [8, 12, 14]. If we additionally assume the monotonic treatment effect condition, then we tautologically identify the direction. As we show in the Appendix, this monotonic treatment effect condition can also provide a bound on the minimum effect of continuous treatment: for example, we may infer that $\left| {{\text{E}}\left[ {Y_{1}^{{a_{0} = 1,a_{1} = 1}} } \right] - {\text{E}}\left[ {Y_{1}^{{a_{0} = 0,a_{1} = 0}} } \right]} \right| \ge \left| {{\text{E}}\left[ {Y_{1} |Z = 1} \right] - {\text{E}}\left[ {Y_{1} |Z = 0} \right]} \right|,$ which aligns with the common statement that the intention-to-treat effect estimate in a trial with non-compliance underestimates the per-protocol effect size (but only under the conditions described here!).

While the direction of the effect is not identified under only the instrumental conditions (i), it has been previously argued in the setting of time-fixed treatments that relatively large sources of heterogeneity would need to be present in order for the direction of the proposed instrument’s effect on the outcome to not align with the direction of the effect of the exposure [7]. Future work is needed to explore how heterogeneity in the time-varying treatment setting affects this conclusion.

## Revisiting Mendelian randomization findings of sustained treatment strategies

To put the above results in context, we revisit two sets of canonical Mendelian randomization results: studies of the causal effects of (i) C-reactive protein (CRP) and (ii) alcohol consumption on risk of cardiovascular disease.

Several Mendelian randomization studies have found null associations between genetic variants related to CRP levels (e.g., variants in the *CRP* gene) and cardiovascular disease [15–17]. Under the condition (i) that the proposed instruments in these studies are indeed instruments, these null associations provide no evidence against any of the causal null hypotheses summarized here. That is, under condition (i), a null association between the genetic variants and cardiovascular disease is consistent with CRP having no effect on cardiovascular disease, but it is also consistent with a non-null causal effect.

Several Mendelian randomization studies have found non-null associations between genetic variants related to alcohol consumption (e.g., variants in the *ADH1B* or *ALDH2* genes) and cardiovascular disease [18–20]. Under condition (i) that the proposed instruments in these studies are indeed instruments, these associations provide evidence against the joint sharp null hypothesis. That is, we would conclude that, for at least one person in the study population, changing alcohol consumption levels by some (unspecified) amount at some (unspecified) point in time would affect cardiovascular disease risk. Evidence against the sharp null hypothesis may be a useful step in the scientific process, but this evidence alone is agnostic to whether specific interventions on alcohol consumption in a population would have beneficial or detrimental effects. For example, testing the joint sharp null hypothesis does not tell us whether reducing alcohol consumption in everybody across the life-course would have a joint non-null effect (i.e., a component of the joint average null), or whether changing alcohol consumption at a certain point in the life-course has an effect in one or more individuals (i.e., a non-joint sharp null).

If we additionally are willing to assume a monotonic treatment effect of alcohol consumption on cardiovascular disease risk (which may not be plausible in this setting), the observed associations in Mendelian randomization studies could also provide evidence against the joint average causal null. Collectively, the publications on alcohol consumption tend to draw further conclusions about the direction of the causal effect. For example, one meta-analysis of Mendelian randomization studies [20] states that “reduction of alcohol consumption, even for light to moderate drinkers, is beneficial for cardiovascular health.” Such conclusions rest upon further causal assumptions, and the biologic plausibility of these assumptions (in addition to the instrumental variable assumptions) needs to be carefully weighed on a case-by-case basis [11].

## Discussion

We have shown that having an instrumental variable is insufficient to test many versions of causal null hypotheses for time-varying exposures, and at best provides evidence concerning a specific joint sharp causal null hypothesis that a change in the exposure at any time would have no effect on the outcome for all individuals. We further described assumptions that, in conjunction with the instrumental variable conditions, allow us to test other sharp and average causal null hypotheses. Our results have important implications for the reporting and interpretation of many Mendelian randomization studies and, more generally, of any study leveraging the instrumental variable assumptions to study sustained treatment strategies, including using an intention-to-treat analysis in a randomized trial to inform our understanding of per-protocol effects.

Throughout this paper, we have ignored statistical considerations about “testing of a null hypothesis.” Dichotomized *p* values are often used to make decisions about whether a hypothesis (including the causal null hypothesis) is true. Such use of *p* values is incorrect, as many authors have demonstrated [21–23], and discouraged by the American Statistical Association [24]. In addition to these concerns, a proper use and interpretation of statistical tests requires that the effect of interest is defined. For example, power calculations have been described for Mendelian randomization studies previously [25–27] primarily if not only for time-fixed treatments. However, any discussion of power for sustained treatment strategies would need to specify the causal contrasts under study.

Finally, there is a discrepancy between emphasizing the use of instrumental variables for hypothesis *testing* only and epidemiologists’ more typical goal of *estimating* causal effects [21–24]. Many epidemiology journals that publish Mendelian randomization or other instrumental variable analyses prefer (or insist upon) effect size estimation regardless of the study design or analysis used. This paper attempts to clarify what can and cannot be tested with an instrumental variable, but does not address the larger issue of whether or how null testing should be conducted.

## Electronic supplementary material

Below is the link to the electronic supplementary material.

Supplementary material 1 (PDF 153 kb)
