# Supplementary material for: Causal null hypotheses of sustained treatment strategies: What can be tested with an instrumental variable?
Source: Eur J Epidemiol. 2018 May 2;33(8):723–8. doi: 10.1007/s10654-018-0396-6 (PMC6061140; doi:10.1007/s10654-018-0396-6)
Supplement: Supplementary file 1 — Supplementary material 1 (PDF 153 kb) [file 10654_2018_396_MOESM1_ESM.pdf]

Appendix: Causal null hypotheses of sustained treatment strategies

SA Swanson, JA Labrecque, MA Hernán

The below results rely on combinations of the following assumptions:

$$Z \coprod Y_0^{a_0}; \tag{A1}$$

$$Z \coprod Y_k^{\overline{a_k}}; \tag{A2}$$

$$Y_0^{a_0} \leq Y_0^{a_0'} \text{ if } a_0 < a_0' \text{ for all subjects and all } a_0, a_0' \in Supp(A_0); \tag{A3}$$

$$Y_k^{\overline{a_k}} \leq Y_k^{\overline{a_k'}} \text{ for any } \overline{a_k} \neq \overline{a_k'} \in Supp(A_k) = [\overline{0}, \overline{1}] \text{ such that } a_j \leq a_j',\ j \in \{1, ..., k\}. \tag{A4}$$

Theorem 1. *Under (A1), suppose the sharp time-fixed causal null holds:*

$$Y_0^{a_0} = Y_0^{a_0'} \textit{ for all subjects and all } a_0, a_0' \in Supp(A_0).$$

Then we have:

$$\mathrm{E}[Y_0|Z = z] = \mathrm{E}[Y_0|Z = z'] \textit{ for all } z, z' \in Supp(Z).$$

Proof. For any $z, z' \in Supp(Z)$ and any $a_0 \in Supp(A_0)$, we have:

$$\mathrm{E}[Y_0^{a_0}] = \mathrm{E}[Y_0^{a_0}|Z = z] = \mathrm{E}[Y|Z = z]$$

$$\mathrm{E}[Y_0^{a_0}] = \mathrm{E}[Y_0^{a_0}|Z = z'] = \mathrm{E}[Y|Z = z']$$

where the first equality in each expression follows from (A1) and the second follows from the sharp time-fixed causal null and consistency. It immediately follows that

$$\mathrm{E}[Y_0|Z = z] = \mathrm{E}[Y_0|Z = z'] \text{ for all } z, z' \in Supp(Z).$$

□

Note: the logic of this proof could similarly apply for Y_k and a single treatment at time $j < k$ if $Z \coprod Y_k^{a_j}$ and an analogous sharp time-fixed causal null regarding $Y_k^{a_j} = Y_k^{a'_j}$. However, this particular condition $Z \coprod Y_k^{a_j}$ will not be reasonable if Z affects treatment at other times besides time j and treatment at other times besides time j can affect Y_k.

Theorem 2. *Under (A2), suppose the sharp joint causal null holds:*

$$Y_k^{\overline{a_k}} = Y_k^{\overline{a'_k}} \text{ for all subjects and all } \overline{a_k}, \overline{a'_k} \in Supp(\overline{A_k}).$$

Then we have:

$$\text{E}[Y_k|Z = z] = \text{E}[Y_k|Z = z'] \text{ for all } z, z' \in Supp(Z).$$

Proof. For any $z, z' \in Supp(Z)$ and any $\overline{a_k} \in Supp(\overline{A_k})$, we have:

$$\text{E}[Y_k^{\overline{a_k}}] = \text{E}[Y_k^{\overline{a_k}}|Z = z] = \text{E}[Y|Z = z]$$

$$\text{E}[Y_k^{\overline{a_k}}] = \text{E}[Y_k^{\overline{a_k}}|Z = z'] = \text{E}[Y|Z = z']$$

where the first equality in each expression follows from (A2) and the second follows from the sharp joint causal null and consistency. It immediately follows that

$$\text{E}[Y_k|Z = z] = \text{E}[Y_k|Z = z'] \text{ for all } z, z' \in Supp(Z).$$

□

Theorem 3. *Under (A1) and (A3), suppose the average time-fixed causal null holds:*

$$\text{E}[Y_0^{a_0}] = \text{E}[Y_0^{a'_0}] \text{ for all } a_0, a'_0 \in Supp(A_0).$$

Then we have:

$$\text{E}[Y_0|Z = z] = \text{E}[Y_0|Z = z'] \text{ for all } z, z' \in Supp(Z).$$

Proof. Define $a_{min} = \min(Supp(A_0))$ and $a_{max} = \max(Supp(A_0))$. For any $z, z' \in Supp(Z)$, we have:

$$\text{E}[Y_0^{a_{min}}] = \text{E}[Y_0^{a_{min}}|Z = z] \leq \text{E}[Y|Z = z]$$

$$\text{E}[Y_0^{a_{min}}] = \text{E}[Y_0^{a_{min}}|Z = z'] \leq \text{E}[Y|Z = z']$$

$$\text{E}[Y_0^{a_{max}}] = \text{E}[Y_0^{a_{max}}|Z = z] \geq \text{E}[Y|Z = z]$$

$$\text{E}[Y_0^{a_{max}}] = \text{E}[Y_0^{a_{max}}|Z = z'] \geq \text{E}[Y|Z = z']$$

where the first equality in each expression follows from (A1) and the inequality follows from (A3). Because $\text{E}[Y_0^{a_{min}}] = \text{E}[Y_0^{a_{max}}]$ under the average time-fixed causal null, it immediately follows that

$$\text{E}[Y_0|Z = z] = \text{E}[Y_0|Z = z'] \text{ for all } z, z' \in Supp(Z).$$

□

Theorem 4. *Under (A2) and (A4), suppose the average joint causal null holds:*

$$\mathrm{E}[Y_k^{\overline{a_k}}] = \mathrm{E}[Y_k^{\overline{a'_k}}] \text{ for all } \overline{a_k}, \overline{a'_k} \in Supp(\overline{A_k})$$

where $Supp(\overline{A_k}) = [\overline{0}, \overline{1}]$*. Then we have:*

$$\mathrm{E}[Y_k|Z = z] = \mathrm{E}[Y_k|Z = z'] \text{ for all } z, z' \in Supp(Z).$$

Proof. For any $z, z' \in Supp(Z)$, we have:

$$\mathrm{E}[Y_k^{\overline{a_k}=\overline{0}}] = \mathrm{E}[Y_k^{\overline{a_k}=\overline{0}}|Z = z] \leq \mathrm{E}[Y|Z = z]$$

$$\mathrm{E}[Y_k^{\overline{a_k}=\overline{0}}] = \mathrm{E}[Y_k^{\overline{a_k}=\overline{0}}|Z = z'] \leq \mathrm{E}[Y|Z = z']$$

$$\mathrm{E}[Y_k^{\overline{a_k}=\overline{1}}] = \mathrm{E}[Y_k^{\overline{a_k}=\overline{1}}|Z = z] \geq \mathrm{E}[Y|Z = z]$$

$$\mathrm{E}[Y_k^{\overline{a_k}=\overline{1}}] = \mathrm{E}[Y_k^{\overline{a_k}=\overline{1}}|Z = z'] \geq \mathrm{E}[Y|Z = z']$$

where the equality in each expression follows from (A2) and the inequality follows from (A4). Because $\mathrm{E}[Y_k^{\overline{a_k}=\overline{0}}] = \mathrm{E}[Y_k^{\overline{a_k}=\overline{1}}]$ under the average joint causal null, it immediately follows that

$$\mathrm{E}[Y_k|Z = z] = \mathrm{E}[Y_k|Z = z'] \text{ for all } z, z' \in Supp(Z).$$

□

Note: A corollary of Theorem 4 is that (A2) and (A4) imply that the average causal effect under continuous treatment vs. continuous no treatment must be greater than or equal to the intention-to-treat association when we have a binary instrument Z. It follows immediately from (A4) that the average causal effect is non-negative, so we only need to show that this average causal effect is not between zero and the intention-to-treat association. The latter can be proven readily by contradiction. (Of course if we reversed the direction of the monotonic treatment effect condition (A4) then under similar logic we would likewise conclude that the absolute value of the average causal effect under continuous treatment vs. continuous no treatment must be greater than or equal to the absolute value of the intention-to-treat association when we have a binary instrument Z.)
